# Supplementary material for: Cause of Death in Heart Failure Based on Etiology: Long-Term Cohort Study of All-Cause and Cardiovascular Mortality
Source: J Clin Med. 2022 Jan 31;11(3):784. doi: 10.3390/jcm11030784 (PMC8837120; doi:10.3390/jcm11030784)
Supplement: Supplementary file 1 [file jcm-11-00784-s001.zip › jcm-1489493-supplementary.pdf]

Supplementary Material

Table S1. Cox regression multivariable analysis for all-cause death and for cardiovascular mortality in patients with NT-proBNP > 1000 ng/L at admission.

	Unadjusted Analysis						Adjusted Analysis *					
	All-Cause Death			Cardiovascular Death †			All-Cause Death			Cardiovascular Death †		
	**HR**	**95%CI**	***p*-value**	**HR**	**95%CI**	***p*-value**	**HR**	**95%CI**	***p*-value**	**HR**	**95%CI**	***p*-value**
IHD	1			1			1			1		
DCM	0.49	0.37–0.64	<0.001	0.51	0.39–0.66	<0.001	0.62	0.47–0.82	<0.001	0.59	0.45–0.78	<0.001
HHD	0.91	0.71–1.17	0.454	0.87	0.68–1.11	0.26	0.88	0.68–1.14	0.35	0.84	0.64–1.09	0.18
ACM	0.34	0.20–0.55	<0.001	0.37	0.23–0.60	<0.001	0.64	0.38–1.06	0.08	0.58	0.36–0.94	0.03
DICM	0.36	0.17–0.77	0.008	0.32	0.16–0.65	0.002	0.66	0.31–1.42	0.29	0.48	0.24–0.98	0.04
VHD	1.09	0.85–1.40	0.5	0.94	0.73–1.21	0.66	0.98	0.75–1.26	0.86	0.86	0.65–1.12	0.26
HCM	0.51	0.23–1.14	0.1	0.5	0.23–1.08	0.08	0.91	0.40–2.06	0.82	0.59	0.26–1.36	0.22

* Adjusted for heart failure duration, New York Heart Association class III-IV, diabetes, anemia, renal insufficiency, chronic obstructive pulmonary disease and peripheral arteriopathy; † Using Fine & Gray competing risk; ACM, alcoholic cardiomyopathy; CI: confidence interval; DCM, dilated cardiomyopathy; DICM, drug-induced cardiomyopathy; HCM, hypertrophic cardiomyopathy; HHD, hypertensive heart disease; HR: hazard ratio; IHD: ischemic heart disease; VHD, valvular heart disease.

Table S2. Cox regression multivariable analysis for all-cause death and for cardiovascular mortality in patients with NYHA III-IV symptoms at admission.

	**Unadjusted Analysis**						**Adjusted Analysis ***					
	**All-Cause Death**			**Cardiovascular Death†**			**All-Cause Death**			**Cardiovascular Death †**		
	**HR**	**95%CI**	***p*-value**	**HR**	**95%CI**	***p*-value**	**HR**	**95%CI**	***p*-value**	**HR**	**95%CI**	***p*-value**
IHD	1			1			1			1		
DCM	0.54	0.36–0.80	0.002	0.57	0.38–0.86	0.007	0.78	0.51–1.18	0.245	0.72	0.47–1.09	0.13
HHD	1.03	0.74–1.44	0.84	0.93	0.67–1.28	0.65	1.03	0.72–1.47	0.877	0.96	0.67–1.37	0.81
ACM	0.32	0.12–0.87	0.03	0.4	0.14–1.09	0.08	0.58	0.21–1.60	0.293	0.59	0.23–1.49	0.27
DICM	0.24	0.08–0.77	0.02	0.22	0.07–0.65	0.007	0.5	0.16–1.59	0.242	0.38	0.13–1.10	0.08
VHD	0.99	0.72–1.37	0.95	0.88	0.63–1.22	0.46	1.11	0.78–1.57	0.572	0.96	0.67–1.38	0.82
HCM	0.28	0.07–1.14	0.08	0.29	0.09–0.95	0.04	0.54	0.13–2.20	0.392	0.37	0.99–1.35	0.13

* Adjusted for heart failure duration, diabetes, anemia, renal insufficiency, chronic obstructive pulmonary disease and peripheral arteriopathy; † Using Fine & Gray competing risk; ACM, alcoholic cardiomyopathy; CI: confidence interval; DCM, dilated cardiomyopathy; DICM, drug-induced cardiomyopathy; HCM, hypertrophic cardiomyopathy; HHD, hypertensive heart disease; HR: hazard ratio; IHD: ischemic heart disease; VHD, valvular heart disease.

Table S3. Rates of death from non-cardiovascular causes according to the etiology of heart failure.

	IHD		DCM		HHD		ACM		DICM		VHD		HCM	
	**Number**	**% of deaths**	**Number**	**% of deaths**	**Number**	**% of deaths**	**Number**	**% of deaths**	**Number**	**% of deaths**	**Number**	**% of deaths**	**Number**	**% of deaths**
Total non-CV deaths	267	33.8	55	41.4	60	39.7	23	46.0	24	75.0	60	39.2	4	40.0
Cancer	82	10.4	17	12.8	14	9.3	10	20.0	19	59.4	16	10.4	0	0.0
Infection	72	9.1	12	9.0	15	10.0	5	10.0	1	3.1	18	11.7	2	20.0
Respiratory	28	3.5	11	8.3	6	4.0	3	6.0	1	3.1	4	2.6	0	0.0
GI	14	1.8	2	1.5	4	2.7	4	8.0	1	3.1	5	3.2	0	0.0
Trauma	9	1.1	2	1.5	2	1.3	1	2.0	0	0.0	5	3.2	0	0.0
Declining	41	5.2	4	3.0	13	8.7	0	0.0	1	3.1	5	3.2	0	0.0
Other	21	2.7	7	5.3	6	4.0	0	0.0	1	3.1	8	5.2	2	20.0

ACM, alcoholic cardiomyopathy; CV, cardiovascular; DCM, dilated cardiomyopathy; DICM, drug-induced cardiomyopathy; GI, gastrointestinal; HCM, hypertrophic cardiomyopathy; HHD, hypertensive heart disease; IHD: ischemic heart disease; VHD, valvular heart disease.
